# Supplementary material for: A Critical View of the Application of the APEX Software (Aqueous Photochemistry of Environmentally-Occurring Xenobiotics) to Predict Photoreaction Kinetics in Surface Freshwaters
Source: Molecules. 2019 Dec 18;25(1):9. doi: 10.3390/molecules25010009 (PMC7017383; doi:10.3390/molecules25010009)
Supplement: Supplementary file 1 [file molecules-25-00009-s001.zip › 3.2.4_gcc-4.4.0.zip!/3.2.4_gcc-4.4.0/3.2.4_gcc-4.4.0/doc/octave/html/interpreter/Array-and-Sparse-Differences.html]

Array and Sparse Differences - Untitled


Next: Creating Sparse Matrices in Oct-Files,
Up: Sparse Matrices in Oct-Files


---

##### A.1.6.1 The Differences between the Array and Sparse Classes

The number of elements in a sparse matrix is considered to be the number
of non-zero elements rather than the product of the dimensions. Therefore

```
     SparseMatrix sm;
     ...
     int nel = sm.nelem ();
```

returns the number of non-zero elements. If the user really requires the
number of elements in the matrix, including the non-zero elements, they
should use `numel` rather than `nelem`. Note that for very
large matrices, where the product of the two dimensions is larger than
the representation of an unsigned int, then `numel` can overflow.
An example is `speye(1e6)` which will create a matrix with a million
rows and columns, but only a million non-zero elements. Therefore the
number of rows by the number of columns in this case is more than two
hundred times the maximum value that can be represented by an unsigned int.
The use of `numel` should therefore be avoided useless it is known
it won't overflow.

Extreme care must be take with the elem method and the "()" operator,
which perform basically the same function. The reason is that if a
sparse object is non-const, then Octave will assume that a
request for a zero element in a sparse matrix is in fact a request
to create this element so it can be filled. Therefore a piece of
code like

```
     SparseMatrix sm;
     ...
     for (int j = 0; j < nc; j++)
       for (int i = 0; i < nr; i++)
         std::cerr << " (" << i << "," << j << "): " << sm(i,j)
                   << std::endl;
```

is a great way of turning the sparse matrix into a dense one, and a
very slow way at that since it reallocates the sparse object at each
zero element in the matrix.

An easy way of preventing the above from happening is to create a temporary
constant version of the sparse matrix. Note that only the container for
the sparse matrix will be copied, while the actual representation of the
data will be shared between the two versions of the sparse matrix. So this
is not a costly operation. For example, the above would become

```
     SparseMatrix sm;
     ...
     const SparseMatrix tmp (sm);
     for (int j = 0; j < nc; j++)
       for (int i = 0; i < nr; i++)
         std::cerr << " (" << i << "," << j << "): " << tmp(i,j)
                   << std::endl;
```

Finally, as the sparse types aren't just represented as a contiguous
block of memory, the `fortran_vec` method of the `Array<T>`
is not available. It is however replaced by three separate methods
`ridx`, `cidx` and `data`, that access the raw compressed
column format that the Octave sparse matrices are stored in.
Additionally, these methods can be used in a manner similar to `elem`,
to allow the matrix to be accessed or filled. However, in that case it is
up to the user to respect the sparse matrix compressed column format
discussed previous.
